# How GPs can help young people avoid future self-harm: a qualitative study

**DOI:** 10.3399/BJGP.2024.0209

**Published:** 2024-10-29

**Authors:** Faraz Mughal, Carolyn A Chew-Graham, Ellen Townsend, Christopher J Armitage, Martyn Lewis, Benjamin Saunders

**Affiliations:** School of Medicine, Keele University, Keele, UK; Unit of Academic Primary Care, Warwick Medical School, University of Warwick, Coventry, UK and Department of General Practice and Primary Care, Melbourne Medical School, University of Melbourne, VIC, Australia.; School of Medicine, Keele University, Keele, UK.; School of Psychology, University of Nottingham, Nottingham, UK.; Manchester Centre for Health Psychology, Division of Psychology and Mental Health, School of Health Sciences, University of Manchester, Manchester, UK and NIHR Greater Manchester Patient Safety Research Collaboration, University of Manchester, Manchester, UK.; School of Medicine, Keele University, Keele, UK.; School of Medicine, Keele University, Keele, UK.

**Keywords:** general practitioners, primary health care, qualitative research, self-harm, self-injurious behavior, young adult

## Abstract

**Background:**

Self-harm is a growing problem in young people. GPs are usually the first point of healthcare contact for young people aged 16–25 years, after self-harm. GPs can experience barriers to supporting young people and behaviour change theory can help to understand these, and the influences on, GP behaviour.

**Aim:**

To explore the capabilities, opportunities, and motivations (COM-B model of behaviour) of GPs, and their perceived training needs, to help young people aged 16–25 years avoid future self-harm.

**Design and setting:**

This was a qualitative study of GPs in England.

**Method:**

Semi-structured interviews were conducted with NHS GPs who were purposively sampled. Interviews occurred in 2021. Data were analysed using reflexive thematic analysis and mapped onto the COM-B model domains. The study’s patient and public involvement group supported data analysis.

**Results:**

Fifteen interviews were completed. Four themes were generated. GPs described mixed capabilities, with many feeling they had the physical and psychological skills to support young people to avoid future self-harm, but some felt doing so was emotionally tiring. GPs identified opportunities to better support young people, such as use of electronic consultation tools, but cited lack of time as a concern. GPs reported motivation to help young people, but this can be influenced by their workload. Unmet training needs around communication, knowledge, and optimising safety were identified.

**Conclusion:**

GPs are supported by their practice teams to support young people after self-harm, but a lack of time hinders opportunities to do so. Future effective GP-led interventions may improve GP motivation to support young people after self-harm.

## Introduction

Self-harm, defined as self-injury or poisoning irrespective of intent, is a growing concern in young people.^1^ Around one in four females, and one in 10 males, aged 16–24 years in England have disclosed past self-harm.^2^ In young people, self-harm can be associated with comorbid depression and anxiety, and can lead to poorer educational outcomes, suicide, and mortality.^3^^,^^4^ The risk of suicide rises 50 times in the year after a self-harm episode compared with the people with no coded self-harm episode in their general practice record.^5^

In general practice, coded self-harm episodes in young people in England have risen, and there has been a 68% increase in the annual incidence of self-harm in 13- to 16- year-old females from 2011 to 2014.^4^ Within 1 year of a first self-harm episode, 22% of 10- to 19- year-olds had a further self-harm episode coded in their record.^4^ Poisoning rates were found to be highest in 16- to 18- year-old females and 19- to 24- year-old males, compared with 10- to 15- year-olds for both sexes.^6^ Young people aged 16–24 years commonly present to a GP after self-harm, thus GPs have an important role in self-harm care.^7^^,^^8^

At present, there are no effective interventions that GPs can use with young people to reduce repeat self-harm behaviour.^9^^,^^10^ GPs can experience barriers to supporting young people after self-harm, and behaviour change theory can help to understand these barriers and identify facilitators and strategies for change.^8^^,^^11^ In this study, the behaviour change theory used was the capabilities, opportunities, and motivations model of behaviour (COM-B),^11^ which has been successfully applied in areas such as knee osteoarthritis and chlamydia testing in general practice^12^^,^^13^ to identify determinants of behaviour change and gain a rich understanding of influences on GP behaviour to inform future intervention development. The COM-B model recognises that for a behaviour to occur people must be capable (physical and psychological), have the requisite opportunities (physical and social), and have sufficient motivation (reflective and automatic).^11^

In this study, we aimed to understand the capabilities, opportunities, and motivations of GPs, and their perceived training needs, to help young people aged 16–25 years avoid future self-harm.

## Method

This qualitative study, conceived before the COVID-19 pandemic, is reported according to the Standards for Reporting Qualitative Research.^14^

**Table:** How this fits in

Young people aged 16–25 years who have self-harmed seek help most commonly from GPs in the NHS. Early GP intervention may help prevent repetition of self-harm. There has been little research exploring influences on GP behaviour to help young people avoid future self-harm. GPs explained mixed capabilities and areas of opportunity to support young people. GPs were motivated and have the desire to support young people, but daily workloads have an impact on this. Future interventions need to improve the capabilities, opportunities, and motivations of GPs to support young people to reduce self-harm behaviour.

### Setting and participants

This study was undertaken in England and participants were GPs who worked in NHS general practice. The exclusion criteria were GPs who were in training or retired; worked in non-routine (for example out of hours) NHS general practice; and worked outside the NHS.

### Recruitment and sampling

Participants were recruited through the National Institute for Health and Care Research Local Clinical Research Networks (LCRNs). Four LCRNs were chosen for geographic spread around England: North East and North Cumbria, West Midlands, East of England, and South West Peninsula. These LCRNs shared the study recruitment advert with their research active general practices and interested eligible GPs were emailed a participant information pack by the first author. In addition, the first author emailed the study recruitment poster to the Royal College of General Practitioners Adolescent Health Group.

Purposive sampling was adopted to aim for a broad and diverse sample of participants in gender, age, years qualified, general practice list size, and average number of sessions per week. Participants were told there were no consequences from withdrawing from the study and that participation was voluntary. All participants were offered reimbursement for their time.

### Patient and public involvement

Five young people aged 16–25 years, with previous self-harm, and three parents or carers of young people who had self-harmed, formed the patient and public involvement (PPI) advisory group. The group, across five remote meetings, helped to design the study recruitment poster, inform the interview topic guide, contribute to the analysis, and plan dissemination.

### Data collection

The first author conducted semi-structured interviews to allow for exploration of interview topics, but encouraged discussion of unexpected areas during interviews. A topic guide, informed by the COM-B domains (see Box 1 for definitions), facilitated data collection, and was refined as analysis occurred concurrently (see Box 2 for interview topics). Interviews were undertaken with GPs from 15 different practices from July to December 2021 on Microsoft Teams or via telephone.

**Box 1.** COM-B domains with definitions^11^

**Capability**	Physical capability: physical skill, strength, or stamina Psychological capability: knowledge or psychological skills, strength, or stamina to engage in mental processes
**Opportunity**	Physical opportunity: environmental influences such as time, resources, cues Social opportunity: interpersonal influences, social cues, cultural norms
**Motivation**	Reflective motivation: reflective processes involving plans and evaluations Automatic motivation: automatic processes involving emotional reactions, desires, impulses, inhibitions, reflex responses

*COM-B = capabilities, opportunities, and motivations model of behaviour.*

**Box 2.** Interview topic guide questions

• Are you physically capable to support young people to engage in non-self-harm behaviour? By ‘physically capable’, I mean the physical skill, strength, or stamina to support young people to engage in non-self-harm behaviour.
• Are you psychologically capable to support young people to engage in non-self-harm behaviour? By ‘psychologically capable’ I mean having the knowledge, the cognitive and interpersonal skills, the ability to engage in appropriate memory and decision-making processes?
• Do you have the physical opportunity to support young people to engage in non-self-harm behaviour? By ‘physical opportunity’ I mean sufficient time, reminders, materials?
• Do you have the social opportunity to support young people to engage in non-self-harm behaviour? By ‘social opportunity’ I mean support from practice colleagues and practice staff?
• Do you have the motivation to support young people to engage in non-self-harm behaviour? For example, the desire to do so, the belief you can and will help, the need to do so?
• Do you feel trying to support or teach young people to engage in non-self-harm behaviour is something you do by habit or impulse in practice?
• What are your training needs to help young people not self-harm?

Interviews were digitally recorded, pseudonymised, and transferred to a professional company for verbatim transcription. The first author checked transcripts against audio-recordings for accuracy. Data collection ceased when additional data no longer offered new insights, and thus data saturation was felt to be reached.^15^ A study risk protocol was designed if distress was identified in participants and participants received a ‘Staying Safe Sheet’ before interviews listing sources of support if needed.

Data management adhered to Keele University Standard Operating Procedures and followed data protection principles and regulations.

### Data analysis

Interview data were analysed using reflexive thematic analysis, which places researcher subjectivity central, is theoretically flexible, and keeps findings close to the original data.^16^ The first author led the analysis, where transcripts were read more than once and coded in an iterative and collaborative manner, including onto the COM-B domains, by three authors. Any tensions identified across participant accounts were moved forward in the analysis where appropriate. Codes were ordered into wider categories that informed the generation of candidate themes with associated data segments. Initial candidate themes were presented to and discussed with PPI and community of practice (CoP) advisory groups, and higher-level themes, which mapped onto COM-B domains, were agreed on among all authors. Analysis was conducted in Word documents and the qualitative software program NVivo 12.

### Reflexivity and trustworthiness

The interviewer, the first author, kept field notes after interviews to inform analysis and reflexivity. The first author recognised that as a practising GP his clinical background may have affected his interaction with participants and orientation and interpretation of the data. This was addressed by encouraging the input of the PPI and CoP groups, and co-authors, in the analysis. All authors considered how their professional backgrounds influenced their understanding of the data. Conducting analyses with researchers from diverse professional backgrounds (applied health research, general practice, social science, behaviour change, clinical trials, and psychology) enhanced the credibility and trustworthiness of findings.^17^

## Results

Fifteen interviews were completed, with a mean duration of 38 min. The age range of participants was 32–52 years, and seven were male and eight female. Ten participants were GP partners, three were salaried GPs, and two were sessional GPs. Most GPs were from East of England (*n* = 5), then North East and North Cumbria (*n* = 4), West Midlands (*n* = 3), South West (*n* = 2), and London (*n* = 1). There was broad experience in practice (from 1 to 22 years) among participants, and the number of average clinical sessions per week was from two to 10 sessions. Participant practice list sizes ranged from 1644 to 39 891, and practices were located within Index of Multiple Deprivation deciles of 2–10. Participant characteristics are shown in Table 1.

**Table 1.** Participant characteristics

**ID**	**Age range, years**	**Gender**	**Location**	**Years qualified**	**Employment status**	**Average number of clinical sessions per week**	**Practice list size, *n***	**IMD decile**
1	40–49	Male	West Midlands	20	Partner	9	3009	2
2	30–39	Male	NENC	7	Partner	6	5348	9
3	30–39	Male	NENC	1	Salaried	6	9032	4
4	40–49	Male	West Midlands	11	Partner	10	3627	3
5	40–49	Female	NENC	15	Partner	2	1644	4
6	40–49	Female	East England	12	Partner	3	7432	6
7	40–49	Male	East England	11	Partner	9	14 821	6
8	40–49	Female	NENC	15	Partner	6	3509	3
9	50–59	Female	East England	22	Partner	6	10 836	9
10	30–39	Male	West Midlands	2	Partner	4	7680	7
11	30–39	Female	London	3	Salaried	4	16 227	3
12	40–49	Female	East England	6	Sessional	2	12 760	9
13	30–39	Male	South West	2	Partner	5	6611	7
14	40–49	Female	East England	20	Sessional	6	14 924	10
15	30–39	Female	South West	8	Salaried	4	39 891	8

*ID = identifier. IMD = Index of Multiple Deprivation. NENC = North East and North Cumbria.*

We generated four themes:

- mixed capabilities;
- opportunities in practice;
- motivations; and
- what else do I need?

We present themes below with accompanying data extracts and participant identifiers. The risk protocol was not activated during this study.

### Mixed capabilities

In this theme, GPs described varied physical and psychological capabilities to help young people avoid future self-harm. When asked if they felt they were physically capable (skills, strength, and stamina – see Box 2), most participants described having the physical strength (being physically strong) and stamina to support young people, aged 16–25 years, to avoid self-harm:

*‘I’m fit and active and without any medical problems, not on any regular medications or anything at all and so I can cope ... with the long hours and stress ... I think I should be able to cope.’*

(GP04, male [M])

*‘I feel physically able, and young people give you a bit of energy as well, so I’m always full of energy when I see them.’*

(GP11, female [F])

Some GPs suggested they could improve their skills to help young people not self-harm in the future and appeared willing to do so:

*‘I think probably the skill – physical skills – I think that’s the least well- honed because you know I’m not a CBT* [cognitive–behaviour therapy] *practitioner. I’m not a mindfulness coach … I could absolutely be upskilled in that.’*

(GP13, M)

*‘The skills, I always feel like I could do better and I’m forever reviewing any new apps or guidance or anything coming in to see would that be better placed to be able to share … they deserve the best evidence- based interventions.’*

(GP14, F)

Nearly all GPs felt they had the knowledge, and cognitive and interpersonal skills, to engage in mental processes to support young people to avoid self-harm:

*‘I personally think I do have those skills; I think a lot of the skills are sort of generic GP skills, you know good consultation techniques, shared decision making, respondent to cues, all the things we get taught right at the start.’*

(GP05, F)

*‘I feel interpersonal skills-wise I’m relatively comfortable … it would be something I relish … come up with a nice little management plan for the patient or something they can go away with. You know, having felt that somebody has not only listened but has given them something they can do to try and help.’*

(GP10, M)

A few participants stated they had undertaken training that enhanced aspects of their psychological capability such as psychological strength and stamina and therefore felt more able to help young people prevent self-harm:

*‘Yes, I do feel I’m psychologically capable of doing it. I’ve done quite a bit of training. I’ve also undertaken a coaching course which required quite a lot of work on myself in some ways … to help someone else, you need to not project your own stuff on them.’*

(GP12, F)

Other GPs described managing self-harm in young people to be emotionally tiring, especially in the context of perceiving young people not wanting to discuss self-harm, and the limited access to specialist support for self-harm, which leads to the GP themselves holding the risk of potential further self-harm or suicide in practice:

*‘Psychologically it can be very draining … like the parents bringing them in and actually they don’t want to be there talking to you about it. So that can be very difficult, and you want to be able to support them, but you can’t … And sometimes it’s frustrating … you refer it on to CAMHS* [Child and Adolescent Mental Health Services] *and you speak to the patient 2 weeks after and they still haven’t been in contact and then you’re left feeling that you’re carrying this risk in the community when it’s not appropriate and what do you do then? So that can be very difficult.’*

(GP11, F)

One participant mentioned how their ability to be sympathetic and show compassion, which can be associated with psychological stamina, is affected by a long day in practice but with experience they had developed mechanisms to remain able to provide safe care:

*‘If it has been a long day, I definitely get a bit of sort of compassion fatigue towards the end of the day, but I think everybody does. I’ve kind of learnt to manage it.’*

(GP15, F)

### Opportunities in practice

Some GPs reflected on physical opportunities to incorporate in-consultation tools in their daily work, such as electronic patient record reminders or text messaging software, to help young people not engage in future self-harm:

*‘We’ve got so much more access to resources than we’ve ever had before, we’ve got Accurx* [electronic platform where patients and NHS staff can communicate]*, we can text links, we can text worksheets … and if whatever intervention we could be trained in I’m sure there’d be some associated resources that could come along with that.’*

(GP10, M)

Many participants identified that a lack of time and materials in the consultation reduced their opportunity to support young people:

*‘Well time is always an issue, obviously you have to make the time if you feel that you can actually add value at that point in that consultation.’*

(GP09, F)

*‘In an actual consultation, I would say there’s probably very little information that I could access.’*

(GP12, F)

Other GPs described how they offered longer consultations with young people who had self-harmed or organised a follow-up appointment at short notice when appropriate, highlighting the ability to be flexible in their clinical work:

*‘I used to try and block two slots if I had someone coming in with their parents because I’d have one slot to talk to them and one slot to talk to them as a family. Now that we’re doing face to face and telephone, we’ve got more flexibility with our face to faces. We bring them in whenever we want, and we have more time.’*

(GP11, F)

Most GPs felt that they would be supported by practice GP and reception staff to help young people avoid self-harm:

*‘I mean I think in terms of my GP colleagues, like I was just saying actually we’re quite a functional practice, we talk to each other a lot, if we had concerns about patients, we would discuss them.’*

(GP06, F)

*‘I think you know our reception* [staff] *are pretty mental health aware, they’re pretty astute to these things … some of our reception staff have had personal experiences which has heightened their awareness.’*

(GP10, M)

Some GPs stated that they feared loss of continuity for young people who have self-harmed, particularly in larger practices:

*‘It’s quite easy to try and get continuity of care at our practice, so we really kind of encourage that. The problem is the bigger you get, [achieving continuity in practice becomes harder] … I do worry about loss of continuity.’*

(GP07, M)

One GP described how working alongside a mental health link worker improved the support they were able to offer young people in practice because they were able to discuss challenging consultations and share best practice:

*‘Mental health link workers* [and additional practice colleagues] *in* [the] *practice … she is actually in the practice which makes such a difference, because you can actually talk to her, I mean these things are important aren’t they? You know, actually being able to have a discussion with someone when you’ve got concerns.’*

(GP06, F)

### Motivations

Most participants suggested that they do feel motivated to help young people avoid future self-harm and reported feeling driven to do so:

*‘I’d say I’m pretty motivated, we see enough of it that you definitely see cases where you’ve had an impact and that helps motivate you further and I think when you do have the time to do it, it’s very satisfying … I’d say very motivated.’*

(GP15, F)

*‘I think it’s really possible. I have got the motivation. I think we need to, within the NHS, recognise these problems* [self-harm] *early and provide that early onset support without pathologising it, otherwise I think we’re going to end up with a huge problem.’*

(GP12, F)

Many GPs described a feeling of needing to help, and the desire and belief that they can help young people who have self-harmed:

*‘I mean you know, when young people do present with their mental health, you can really make a big difference if you get it right at this stage … I’ve certainly got the desire to do it, personally, and would be very keen to do so.’*

(GP10, M)

*‘A lot of things we do in medicine aren’t because that’s what you’re meant to do, following some guideline and protocol, but because you feel you have to, and this would be a very good example of that.’*

(GP02, M)

A few GPs explained that their motivation was negatively influenced by their daily workload. It was felt that the energy required to support self-harm management was lacking on days when they had a particularly heavy workload:

*‘I think that it is psychologically draining at times. I think it will be dependent on day- to- day. If I look at my morning, we tend to have five free bookable slots before our duty* [on call] *starts. I would be actively not booking somebody in that slot because I would already be slightly distracted by what’s happening ahead of me ... I think you can do a lot to influence an individual, but I think it’s a tiring process.’*

(GP13, M)

When considering automatic motivation (see Box 1 for definition), GPs described varying motivation to impulsively help young people avoid future self-harm. Most participants stated they would immediately have the impulse to support young people:

*‘If someone’s presenting with self- harm then of course you’re not going to brush it under the carpet, you know, you want to help in any which way you can.’*

(GP05, F)

*‘Yeah, definitely. I think it’s hard not to touch on it. I’m aware of time, which is the only limiting thing, but I think instinctively I’m wanting to do it* [help young people avoid self-harm]*, yeah.’*

(GP15, F)

Other GPs mentioned how their motivation was influenced by past encounters and that having a framework to use in the consultation would improve their automatic processes to help young people avoid self-harm:

*‘I think it depends how the first few times go. So, if I get a lot of success, then I would probably, that would feed the cycle, and then I would do a bit more of it.’*

(GP08, F)

### What else do I need?

Most participants stated that they would benefit from communication phrases to use in consultations with young people following self-harm:

*‘What you can actually say to them, how are you going to speak to them, what are the exact words you’re going to use. What are you going to say, because that’s the hardest bit for me … some simple prompts.’*

(GP01, M)

*‘I think GPs need to know obviously why people self- harm and need to know the difference between self- harm and suicidal thoughts. I think we need to know how to explain that to an adolescent in like simple terms or without using stigmatising language because understanding it and explaining it to someone else is different.’*

(GP 11, F)

GPs identified that understanding why a young person has self-harmed, and subsequently discussing self-harm with a young person are two different consultation processes, each requiring specific knowledge and skills. Some participants wanted to know about the function of self-harm in young people, and others suggested being upskilled in brief techniques to use with young people:

*‘Understanding of why self- harm works for the young person … a little bit about DBT* [dialectical behaviour therapy] *and CBT and the importance of reframing and how language can be really important there ... and how to provide a space. I guess you want to introduce a bit of a coaching style to it.’*

(GP12, F)

*‘It’s about knowledge as well and about teaching them* [the] *process of what to do* [when the urge to self-harm occurs] *and I suppose it would be a mini kind of CBT or crisis management that the GP would have to know and then communicate to the patient.’*

(GP03, M)

Some GPs said that online education and learning could enable them to support young people more:

*‘Some online modules about techniques to explain to us what they are, the brief theory behind them and how we can deliver them safely and relatively successfully. That could be useful for all of us, and all GPs are capable of completing you know an hour or two.’*

(GP10, M)

Other GPs felt a need for training on how to handle future risk of self-harm or suicide in young people:

*‘I think the other thing is sometimes it’s difficult to know at what point, from a safety netting point of view, you feel comfortable … I wouldn’t refer a self- harm to a crisis team, you know there’s extremes of self- harming behaviour, so I think maybe a pathway for risk management as well.’*

(GP15, F)

GPs explained how communication prompts, knowledge, and skills around why and how to discuss self-harm, online learning, and how to manage risk in young people were all areas of unmet training need.

## Discussion

### Summary

This study is likely the first, to the authors’ knowledge, to explore GPs’ perspectives on how they can help young people aged 16–25 years avoid self-harm through the lens of behaviour change. GPs described feeling they had the physical strength, stamina, and psychological skills to help young people avoid self-harm, but some GPs felt doing so can be emotionally tiring, which in turn affects their psychological stamina.

Many GPs identified that a lack of time reduces their opportunity to help young people in the consultation, but they felt well supported by their practice teams to do so. GPs stated a strong motivation, desire, and impulse to support young people, but explained that their motivation can be influenced by their workload and past encounters with young people after self-harm. Finally, GPs identified unmet training needs in communication prompts, functions of self-harm, brief techniques to use with young people, online learning, and optimising the future safety of young people.

### Strengths and limitations

This study allowed for a rich understanding of GPs’ views on how they can help young people avoid future self-harm according to their capabilities, opportunities, and motivations. The findings were generated through a recursive and collaborative analysis process and can facilitate future behavioural analyses of how to influence target behaviours specific to GPs in this field. The diversity in participant characteristics led to a varied sample with insights attained from different areas of deprivation. The involvement of the PPI and CoP groups in the study improved the relevance and integrity of findings.

There were, however, several limitations. Participants were likely interested in self-harm in young people and thus views attained are likely not representative of most GPs. One potential limitation of using COM-B-informed interview questions and mapping themes onto COM-B domains is that some COM-B components may not appear to be relevant, and thus COM-B domains may be imposed on the data, rather than generated from the data, such as the physical strength reference within the mixed capabilities theme. There was the chance that the interviewer’s interest in self-harm in young people, including any preconceptions held, influenced the analysis, but the first author attempted to bracket prior assumptions, included PPI group members, and made field notes to mitigate the risk of this.^18^ Participants may have also felt that their clinical practice was being tested and this may therefore have resulted in partial accounts only being gained in interviews.^18^

### Comparison with existing literature

This study advances the literature on how GPs can better support young people, aged 16–25 years, to avoid future self-harm by presenting novel findings centred around the COM-B framework to understand how to influence GPs’ behaviour when managing young people after self-harm. Fox *et al* previously described that GPs lacked confidence when talking to young people about self-harm and felt that consultations with young people and their carers can be challenging.^19^ In this study, GPs did not mention concerns around confidence: most GPs felt they had the psychological capability (skills and knowledge) and motivation to help young people avoid future self-harm, but that a lack of time in the consultation hindered their ability to do so.

We identified that GPs had a training need in relation to keeping young people safe after self-harm and this mirrors the views of Australian GPs.^20^ A recent study that focused on the implementation of national self-harm guidelines by GPs highlighted information and skill needs in areas of communication, self-harm knowledge, and risk assessment, corroborating what we found in this study albeit specific to managing young people aged 16–25 years.^21^ The functions of self-harm in young people aged 16–25 years have recently been identified and this can support addressing knowledge gaps for GPs.^22^

### Implications for research and practice

Unmet training needs about functions of self-harm, communication, and how to optimise the safety of young people after self-harm were identified and thus evidence-based education and skills training may further enhance the capability and motivation of GPs to help young people avoid engaging in self-harm. Training incorporating lived and professional experiences, which can be delivered online, are likely to be more acceptable to GPs. GPs’ psychological capability could be improved by including discussion about how GPs can navigate feelings of emotional fatigue, as our findings indicated. Where appropriate, GPs can consider longer consultation times or double appointments with young people in practice to improve opportunities to help young people after self-harm. Current pressures on NHS general practice may, however, deter GPs from considering this.

Future research can explore how to improve GPs’ capabilities, opportunities, and motivations to help young people avoid self-harm through further behavioural analyses, such as using the theoretical domains framework or behaviour change wheel to gain a more detailed understanding about how to influence certain GP behaviours and to identify intervention types.^11^ In the context of current general practice, there is an urgent need for effective brief GP-led interventions to help young people avoid future self-harm, which will likely enhance the motivation of GPs, and address the intervention evidence gap.

## References

[1] National Institute for Health and Care Excellence (2022). Self-harm: assessment, management and preventing recurrence [NG225].

[2] McManus S, Gunnell D, Cooper C (2019). Prevalence of non-suicidal self-harm and service contact in England, 2000–14: repeated cross-sectional surveys of the general population. Lancet Psychiatry.

[3] Mars B, Heron J, Crane C (2014). Clinical and social outcomes of adolescent self harm: population based birth cohort study. BMJ.

[4] Morgan C, Webb RT, Carr MJ (2017). Incidence, clinical management, and mortality risk following self harm among children and adolescents: cohort study in primary care.. BMJ.

[5] Carr MJ, Ashcroft DM, Kontopantelis E (2017). Premature death among primary care patients with a history of self-harm. Ann Fam Med.

[6] Tyrrell EG, Kendrick D, Sayal K, Orton E (2018). Poisoning substances taken by young people: a population-based cohort study. Br J Gen Pract.

[7] Marchant A, Turner S, Balbuena L (2020). Self-harm presentation across healthcare settings by sex in young people: an e-cohort study using routinely collected linked healthcare data in Wales, UK. Arch Dis Child.

[8] Mughal F, Troya MI, Dikomitis L (2020). Role of the GP in the management of patients with self-harm behaviour: a systematic review.. Br J Gen Pract.

[9] Witt KG, Hetrick SE, Rajaram G (2021). Psychosocial interventions for self-harm in adults. Cochrane Database Syst Rev.

[10] Witt KG, Hetrick SE, Rajaram G (2021). Interventions for self-harm in children and adolescents. Cochrane Database Syst Rev.

[11] Michie S, Atkins L, West R (2014). The Behaviour Change Wheel.

[12] Egerton T, Nelligan RK, Setchell J (2018). General practitioners’ views on managing knee osteoarthritis: a thematic analysis of factors influencing clinical practice guideline implementation in primary care. BMC Rheumatol.

[13] McDonagh LK, Saunders JM, Cassell J (2018). Application of the COM-B model to barriers and facilitators to chlamydia testing in general practice for young people and primary care practitioners: a systematic review. Implement Sci.

[14] O’Brien BC, Harris IB, Beckman TJ (2014). Standards for reporting qualitative research: a synthesis of recommendations. Acad Med.

[15] Saunders B, Sim J, Kingstone T (2018). Saturation in qualitative research: exploring its conceptualization and operationalization. Qual Quant.

[16] Braun V, Clarke V (2022). Thematic analysis: a practical guide.

[17] Henwood KL, Pidgeon NF (1992). Qualitative research and psychological theorizing. Br J Psychol.

[18] Chew-Graham CA, May CR, Perry MS (2002). Qualitative research and the problem of judgement: lessons from interviewing fellow professionals. Fam Pract.

[19] Fox F, Stallard P, Cooney G (2015). GPs role identifying young people who self-harm: a mixed methods study. Fam Pract.

[20] Bellairs-Walsh I, Byrne SJ, Bendall S (2021). Working with young people at risk of suicidal behaviour and self-harm: a qualitative study of Australian general practitioners’ perspectives. Int J Environ Res Public Health.

[21] Leather JZ, Keyworth C, Kapur N (2022). Examining drivers of self-harm guideline implementation by general practitioners: A qualitative analysis using the theoretical domains framework. B J Health Psychol.

[22] Mughal F, Chew-Graham CA, Babatunde OO (2023). The functions of self-harm in young people and their perspectives about future general practitioner-led care: a qualitative study. Health Expect.

